# WeChat-based intervention to support breastfeeding for Chinese mothers: protocol of a randomised controlled trial

**DOI:** 10.1186/s12911-020-01322-8

**Published:** 2020-11-19

**Authors:** Li Tang, Andy H. Lee, Colin W. Binns, Lian Duan, Yi Liu, Chunrong Li

**Affiliations:** 1 Chengdu Women’s and Children’s Central Hospital, School of Medicine, University of Electronic Science and Technology of China, Chengdu, 611731 China; 2 School of Public Health, Curtin University, Perth, Australia; 3 Chengdu Jinjiang Maternity and Child Health Hospital, Chengdu, China

**Keywords:** Exclusive breastfeeding, Smart phone, WeChat, Randomised controlled trial

## Abstract

**Background:**

Exclusive breastfeeding for the first 6 months of life is the optimal way to feed infants. However, recent studies suggest that exclusive breastfeeding rates in China remain low and are well below the recommended target. There has been evidence that a lack of awareness of, or exposure to, breastfeeding information is associated with poor breastfeeding practices. WeChat, the most widely used social networking platform in China, has shown some potential to promote health behaviours. We thus hypothesised that a breastfeeding intervention program delivered via WeChat would achieve at least a 10% increase in exclusive breastfeeding prevalence at 6 months compared to the control group.

**Methods:**

A two-arm, parallel, multicentre randomised controlled trial of 1000 pregnant women will be conducted at four maternity hospitals of Chengdu, China. Eligible women who consent to participate in the trial will be recruited at 28–30 weeks of gestation, and randomly allocated to either the intervention group (participants receive breastfeeding-related information from WeChat) or the control group (participants receive non-breastfeeding information from WeChat) using a central randomisation system on a 1:1 ratio at each participating site. The primary outcomes are exclusive breastfeeding rate and full breastfeeding rate at 6 months postpartum. All randomised participants will be included in the outcome analyses with missing data being imputed based on the best-case and worst-case scenarios. Multilevel mixed regression models will be used in the primary analyses to assess the effectiveness of intervention program on the breastfeeding rates.

**Discussion:**

This trial uses the most widely used social media program as a means of delivering messages to mothers to increase exclusive breastfeeding in China. Increasing exclusive breastfeeding will contribute to meeting the health and environmental goals of the Sustainable Development Guidelines.

*Trial registration* ClinicalTrials.gov, NCT04499404. Registered 5 August 2020—Retrospectively registered, https://clinicaltrials.gov/show/NCT04499404

## Background

Breastfeeding is known to provide both short-term and long-term benefits to infants, mothers and the society in general [1, 2]. Both the World Health Organization (WHO) and the Chinese Ministry of Health recommend that infants should be exclusively breastfed for the first 6 months of life to achieve optimal growth, development and health [3]. Exclusively breastfed infants have a reduced risk of infectious diseases such as diarrhoea, and pneumonia, and are less likely to develop obesity and diabetes in later life [4–6]. Many studies have also confirmed that breastfeeding is associated with improved performance in intelligence tests during childhood and adolescence [7].

Despite the majority of Chinese infants are initially breastfed for at least one feed, the exclusive breastfeeding rate at 6 months falls significantly below ideal levels [8, 9], with infant formula being widely used in both rural and urban China [10, 11]. According to a report from UNICEF, only 20.8% of Chinese infants were exclusively breastfed within the first 6 months of life in 2013 [12]. A recent survey undertaken across China during 2017–2018 shows that the rate of exclusive breastfeeding remains low, with less than 30% of infants aged 0–6 months being exclusively breastfed [13]. Given the high prevalence of infant formula use and related health problems, every effort is needed to improve breastfeeding practices in China to meet the recommended target.

Previous research has identified a wide range of sociodemographic, biophysical, psychosocial and cultural factors associated with breastfeeding practices, including a lack of awareness to information that promote and supports breastfeeding [14, 15]. Education and improving access to skilled lactation counselling have been shown to increase exclusive breastfeeding rates by 90% [16].

Many studies have suggested to deliver breastfeeding education and related information via the internet or mobile phone short messages service (SMS) to improve breastfeeding outcomes. For instance, in a quasi-experimental cohort study of 582 women in Shanghai, the median duration of exclusive breastfeeding and exclusive breastfeeding rate at 6 months postpartum were significantly increased in the intervention group who received infant feeding messages delivered by SMS [17]. A systematic review and meta-analysis showed that women who received antenatal interventions using SMS/mobile phone were more likely to initiate breastfeeding within 1 hour after childbirth and less likely to stop exclusive breastfeeding before 6 months compared to women who received routine care [18].

Alongside the rapid development of internet and information technology, social media and smartphones have been widely used in health education research and have shown potential in improving health behaviours [19, 20]. WeChat, a free smartphone application, is the most frequently and widely used social networking platform in China. According to a report from Tencent, Inc., the developer of WeChat, its monthly-active-user reached 1.16 billion in the last quarter of 2019. Besides instant text messaging and free audio/video calls, WeChat provides many other daily living services including mobile payment, browsing and posting information sharing on moments, sending red envelops and pay utility bills [21]. As comparable to Facebook pages, WeChat public accounts can send push notifications to their followers and alert them to new content.

Due to its convenience and availability, WeChat has become a new medium and tool for health information dissemination, with great potential to affect the general public’s health behaviours. Compared to mobile phone SMS, WeChat public accounts are able to deliver health education messages in a cheaper and more visual, enjoyable way. Some studies conducted in China showed that WeChat platform is effective in health promotion interventions [22, 23]. In addition, the use of online health counselling services, including WeChat-based resources, becomes particularly popular under the coronavirus disease 2019 (COVID-19) outbreak in China [24]. However, to our knowledge, no studies have been conducted to investigate the effectiveness of a WeChat-based intervention to promote breastfeeding practices.

We have designed a randomised controlled trial (RCT) for a breastfeeding intervention program using WeChat in urban China to increase exclusive breastfeeding rates. Our hypothesis is that the intervention program will lead to at least a 10% increase in exclusive breastfeeding prevalence at 6 months when compared to the control group.

## Methods

### Study design and setting

A two-arm, parallel RCT of 1000 participants (500 in each arm) will be conducted at four maternity hospitals in Chengdu, China. Chengdu, the capital of Sichuan Province, is located in southwest China with a population of 15.9 million. According to the National Bureau of Statistics, the disposable annual income per capita of urban residents in Chengdu was 42,128 RMB in 2018, slightly higher than the average national level of 39,251 RMB.

**Fig. 1.**
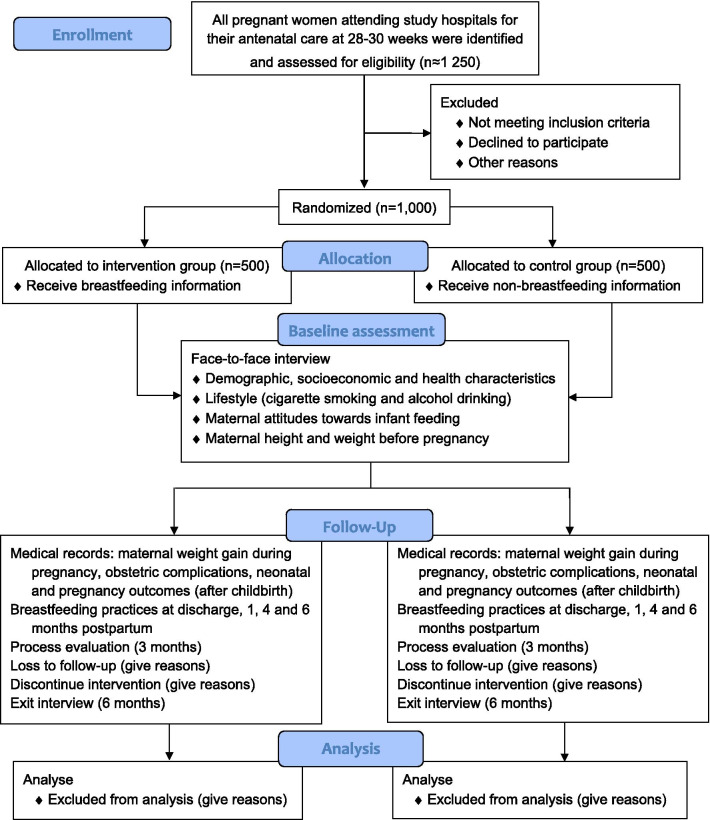
Flow diagram of the RCT

Eligible pregnant women who consent to participate will be recruited from the antenatal clinic of the four maternity hospitals between 28 and 30 weeks of gestation and will be randomly assigned to either the intervention or control group via a central randomisation system on a 1:1 ratio at each participating site. Based on their number of live births in 2018, a total of 300, 300, 200 and 200 women will be recruited, respectively, from Chengdu Women's and Children's Central Hospital, Chengdu Jinjiang Maternity and Child Health Hospital, Chengdu Qingyang Maternal and Child Health Hospital, and Chengdu Wuhou Maternal and Child Health Hospital. Participants recruitment started in June 2020 and is expected to end in May 2021. We followed the Standard Protocol Items: Recommendations for Interventional Trials (SPIRIT) guidelines for developing our trial protocol [25, 26]. The design of the RCT is summarized in Fig. 1.

### Participants and recruitment

Pregnant women meeting the following inclusion criteria will be invited to participate in the study: (1) 18 years or above, (2) own a smartphone, (3) carry a singleton fetus, (4) at a gestational age of 28 to 30 weeks, and (5) have sufficient language skills (i.e. completed secondary school education).

Pregnant women will be excluded from the study if they meet either of the following criteria: (1) have existing medical conditions or pregnancy complications which may inhibit breastfeeding initiation, according to their medical doctor, (2) intend to give birth in health institutes other than the study hospitals.

The aims of the study (i.e. to improve maternal and child health) and participants’ right to withdraw at any time will be explained to potentially eligible women both verbally and via an information sheet. The consent form will be completed and signed by the participants prior to recruitment.

### Randomisation and baseline data collection

Eligible pregnant women consenting to participate in the study will be randomly assigned via a central randomisation system to an intervention group (participants receive breastfeeding-related information from WeChat) or a control group (participants receive non-breastfeeding information from WeChat) at an allocation ratio of 1:1. We will perform block randomisation within each participating hospital, with varying block sizes of 4 or 6.

After randomisation, a trained nurse who is not aware of the group allocation will conduct face-to-face interview of the consented participant using a structured questionnaire (Additional file 1) to collect information on sociodemographic and health characteristics, and lifestyle (cigarette smoking and alcohol drinking). Information on maternal attitudes towards infant feeding will be solicited using the Chinese version of the Iowa Infant Feeding Attitude Scale [27]. Self-reported data on height and weight before pregnancy will also be collected at baseline interview.

### Interventions

**Fig. 2.**
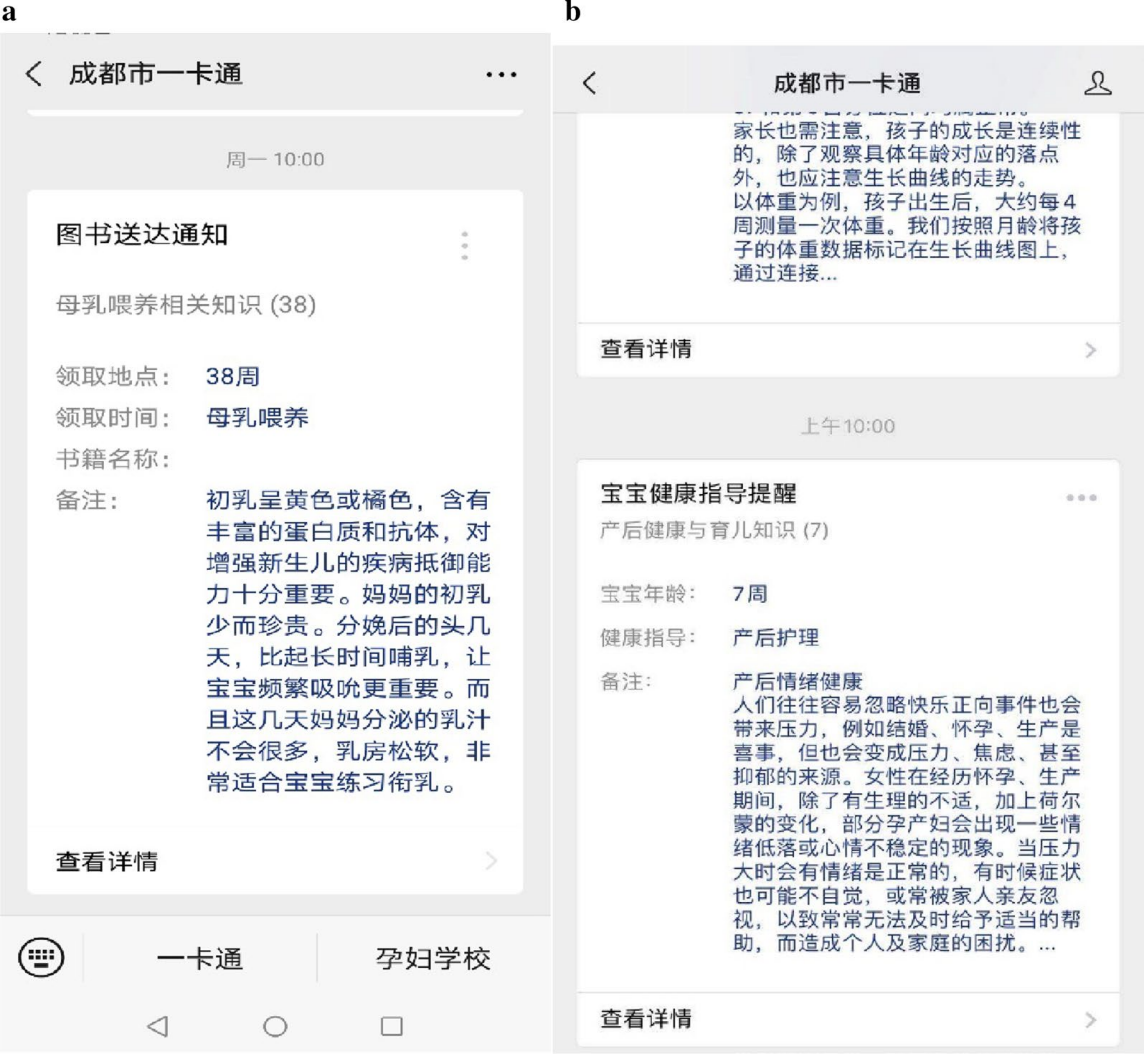
Examples of pushed content (**a** example of pushed content for the intervention group; **b** example of pushed content for the control group). The image depicted in figure is our own.

Immediately after baseline interview, all participants will be asked to scan a Quick Response (QR) code to follow our WeChat public account named ‘Chengdu Maternal and Child Health Information eCard Management System’, which will send push notifications to alert the participants for new content, topics, and frequently asked questions and answers (Fig. 2). In order to minimise potential contamination, the messages are visible to our study participants only and the information forwarding function is disabled. In addition, participants are told not to share the messages with others in ways such as screenshot.

From baseline (30 weeks of gestation) until childbirth, the intervention group participants will receive breastfeeding related messages (one message each time, three times a week) from WeChat, including preparation for breastfeeding after birth and the health benefits of breastfeeding, whereas the control group women will receive messages mainly on topics of healthy lifestyle and nutrition during pregnancy. Before delivery, there are ten messages for each group and the repeat cycle is 3 weeks.

After childbirth, mothers in the intervention group will continue to receive information, which is mainly about breastfeeding, twice a week (one message each time) for 6 months. We will use WeChat to remind mothers about the importance of exclusive breastfeeding, build confidence and motivate them to continue exclusive breastfeeding. Meanwhile, the control group mothers will receive information mainly on routine parental care, such as immunization, safety, and infant growth. The dose and frequency of messages are the same as the intervention group. We developed the intervention content based on literature review, expert consultations and focus group discussion among pregnant and postpartum women. Every participant receives regular prenatal and postpartum maternity services. All the messages are presented in the Additional file 2.

### Formative research focus groups

Two focus groups, one with ten pregnant women and another with ten postpartum mothers, were undertaken to pilot test the intervention content and questionnaires before the implementation of the trial. These research materials have been modified based on the feedbacks to ensure their contents are relevant and appropriate to the target group.

### Follow-up and outcomes

After childbirth, information on maternal weight gain during pregnancy, obstetric complications and neonatal and pregnancy outcomes, such as delivery method, birthweight and infant gender, will be extracted from medical records. Each participant will be interviewed in person by trained nurses at discharge and be interviewed by telephone at 1, 4, and 6 months postpartum to collect detailed information on breastfeeding practices. Information gathered from the participants will be entered into tablet computers during the interviews. Preliminary logic and range checks are built into the data entry system. Data will be saved locally and synchronized to server once the device is connected to an internet connection. The questionnaires used (Additional file 1) are taken from our previous studies conducted in Sichuan Province [28, 29], whose validity has been verified for Chinese mothers.

#### Primary outcomes

The primary outcomes are exclusive breastfeeding rate and full breastfeeding rate at 6 months postpartum. We follow the WHO’s definition of exclusive breastfeeding as “Breastfeeding while giving no other food or liquid, not even water, with the exception of drops or syrups consisting of vitamins, mineral supplements or medicines” [30]. Full breastfeeding is defined as “Infants who are receiving almost all of their nutrients from breast milk but take some other liquids such as water, water-based drinks, oral rehydration solutions, ritual fluids, and drops or syrups” [31].

To obtain information on infant feeding practices at 6 months, mothers will be asked the following two questions: (1) How did you feed your baby in the past 24 h? The response options are: “Breastfeeding only”, “Breastfeeding + water/glucose water/fruit juice”, “Mainly breastfeeding but ‘topping up’ with infant formula”, “Mainly infant formula but also breastfeeding”, and “Infant formula only”; (2) Did you give complementary foods to your baby in the past 24 h? If the response is ‘Yes’, mothers will be asked to provide the name of each complementary food. In our study, complementary feeding is defined as feeding infants with solid foods and liquids other than breastmilk or infant formula [32].

#### Secondary outcomes

The secondary outcomes are: (1) infant’s first feed, (2) exclusive breastfeeding to 4 months postpartum, (3) exclusive breastfeeding to 6 months postpartum, (4) rate of early introduction of complementary feeding before 4 months, and (5) any breastfeeding duration within 6 months postpartum. The definition of any breastfeeding is “The child has received breastmilk (direct from the breast or expressed) with or without other drink, formula or other infant food” [30].

### Blinding

Study coordinators, participants, nurses conducting the interview and statistician performing the data analysis will be masked to the group allocation. Only the investigator who created the randomisation tables is aware of the randomisation assignments.

### Process evaluation

Several components of the program, including its quality, completeness, exposure and satisfaction, participation rate, recruitment, and content, will be evaluated midway of the intervention. Participants’ perceptions of the readability, usefulness, suitability and relevance of the intervention resources will be collected using a brief questionnaire.

### Exit interview

We will randomly select 10 intervention and 10 control participants (10 program completers and 10 non-completers) to participate in the exit interview. The main purpose is to collect information on participants’ opinions of the intervention program and of the messages they obtain in order to further refine and improve the intervention in future. Reasons for withdrawal before completing the project will be solicited from the non-completers. Each interview will take less than 20 min.

### Sample size

Exclusive breastfeeding rates at 6 months postpartum (the primary outcome variable) and the sample size of the study was determined using the formula as follows [33]:

$${\text{n}} = \left\{ {\frac{{{\text{Z}}_{{\alpha /2}} \sqrt {{\text{P}}_{{\text{e}}} \left( {1 - {\text{P}}_{{\text{e}}} } \right)} + {\text{Z}}_{{\beta /2}} \sqrt {{\text{P}}_{1} \left( {1 - {\text{P}}_{1} } \right) + {\text{P}}_{2} (1 - {\text{P}}_{2} )} }}{{{\text{P}}_{1} - {\text{P}}_{2} }}} \right\}^{2}$$

where n is the required sample size for each group; Z_α/2_ and Z_β/2_ are the critical Z values for a given alpha and beta level respectively; P_1_ and P_2_ are the estimated exclusive breastfeeding rates at 6 months in the control and intervention group, respectively; P_e_ = P_1_ × 0.50 + P_2_ × 0.50.

Based on the results of our recent prospective cohort conducted in Chengdu [34], we assume that the exclusive breastfeeding rate for mothers in the control group is P_1_ = 25% at 6 months. In addition, we hypothesize a conservative effect size of 10% improvement in the exclusive breastfeeding rate for the intervention group relative to the control group (P_2_ = 35%). The estimated sample size is 890 (450 per group) under a power of 90% and a two-sided significance level of 5%. Taking into account a loss to follow-up of 10%, the total sample size required at baseline for the RCT is 1000 (500 per group). It was assumed that approximately 20% of women will decline to participate, especially due to the COVID-19 pandemic, we therefore plan to approach about 1250 women at the four hospital antenatal clinics.

### Statistical analysis

Descriptive statistics will be performed to summarise participants’ baseline characteristics and the outcome variables. Independent t-tests or Mann–Whitney U tests will be performed to compare continuous variables and chi-square or Fisher’s exact tests to compare categorical characteristics.

Kaplan–Meier curves and log-rank test will be used to compare breastfeeding duration between the intervention and control groups. The Cox proportion hazards model will be conducted to determine the hazard ratios of exclusive and any breastfeeding cessation, accounting for baseline characteristics and other plausible factors associated with breastfeeding practices. Similar analyses will be applied to analyse the data of complementary foods introduction. Multilevel mixed regression models will be used to assess the effectiveness of intervention program on breastfeeding rates at four and 6 months.

We will follow the intention-to-treat principle with all randomised participants included in the analysis, and the missing data will be imputed based on the best-case and worst-case scenarios. Subgroup analyses will be conducted according to variables of interest.

QSR International NVivo 11 software will be used to perform qualitative analysis. The focus group discussions and in-depth interviews will be transcribed verbatim and inductive thematic analysis will be applied to analysis of the transcripts from process evaluation and the exit interviews.

### Monitoring

We will develop a data monitoring committee (DMC) to monitor conduct and quality of the trial. The DMC includes four independent members with expertise in maternal and infant health, nutrition, biostatistics and trial methodology. The DMC meeting will take place every 6 months.

## Discussion

Although breastfeeding is widely recognized as the best feeding method for infants, the breastfeeding practices in China, in particular exclusive breastfeeding, remain resistant to improvement. The present trial is designed to improve breastfeeding practices of mothers who have a smartphone in urban China. It will be the first study to evaluate the effectiveness of delivering a breastfeeding intervention via WeChat for Chinese mothers.

One main strength of our proposed trial is the use of the widely available and highly convenient tool—WeChat. Every day, more than one billon WeChat users open WeChat at least once, 45 billion messages are sent, and 410 million audio and video calls are made [35]. WeChat is now the most popular social media platform among Chinese. According to our previous research, many mothers in China still have misconceptions on infant feeding and lack true understanding of the health benefits of breastfeeding, both of which largely contribute to the introduction of infant formula within 6 months in China [36]. Delivery of evidence-based educational messages using modern smartphone technology and popular social media networks has the potential to empower mothers to make better informed decisions regarding infant feeding. In addition, the adoption of an RCT design confers a sound basis and an ideal condition for evaluating the intervention effectiveness.

Our study has some limitations. Since we do not have access to the login information of WeChat users, the data on participants’ actual exposure to and uptake of the educational messages will not be available. However, during follow-up, we will ask the mothers to report whether and how often they actually log on WeChat to read the educational messages. In addition, we only follow up mothers to 6 months postpartum due to budget constraint. A longer term of follow-up of 1 year or more is recommended in future studies.

Exclusive breastfeeding provides the most cost-effective intervention for improving the health of infants in the short term and later in life. This trial will use the most widely used social media program as a means of delivering messages to mothers to increase exclusive breastfeeding. Increasing exclusive breastfeeding will contribute to meeting the health and environmental goals of the Sustainable Development Guidelines.

## Supplementary information

**Additional file 1**: Survey questionnaires.

**Additional file 2**: Intervention messages.

## Data Availability

The datasets used and/or analysed during the current study are available from the corresponding author on reasonable request.

## Abbreviations

COVID-19: Coronavirus disease 2019
DMC: Data monitoring committee
QR: Quick response
RCT: Randomised controlled trial
SMS: Short messages service
WHO: World Health Organization
