# Correction to: WeChat-based intervention to support breastfeeding for Chinese mothers: protocol of a randomised controlled trial

**DOI:** 10.1186/s12911-020-01379-5

**Published:** 2021-02-08

**Authors:** Li Tang, Andy H. Lee, Colin W. Binns, Lian Duan, Yi Liu, Chunrong Li

**Affiliations:** 1 Chengdu Women’s and Children’s Central Hospital, School of Medicine, University of Electronic Science and Technology of China, Chengdu, 611731 China; 2 School of Public Health, Curtin University, Perth, Australia; 3 Chengdu Jinjiang Maternity and Child Health Hospital, Chengdu, China

## Correction to: BMC Med Inform Decis Mak (2020) 20:300 10.1186/s12911-020-01322-8

Following publication of the original article [1], it was reported that the contents of Additional file 2 were a duplicate of the files for Additional file 1.

The correct files for Additional file 2 are included in this Correction article and the original article has been updated.

## Supplementary Information

**Additional file 2.** Intervention message.
