# Supplementary material for: WeChat-based intervention to support breastfeeding for Chinese mothers: protocol of a randomised controlled trial
Source: BMC Med Inform Decis Mak. 2020 Nov 19;20:300. doi: 10.1186/s12911-020-01322-8 (PMC7676472; doi:10.1186/s12911-020-01322-8)
Supplement: Supplementary file 1 — Additional file 1: Survey questionnaires. [file 12911_2020_1322_MOESM1_ESM.docx]

Participant ID:

**QUESTIONNAIRE 1 (Baseline interview)**

**PART I: BASIC INFORMATION**

Date of interview: / / (DD/MM/YYYY)

Interviewer code: □□□□

Participant’s name: ___________________________________________

Participant’s date of birth: / / (DD/MM/YYYY)

Gestational age: weeks days

Phone number: _________________________________________

Husband/next of kin’s phone number: ________________________________

**PART II: SOCIO-DEMOGRAPHIC AND ANTHROPOMETRIC CHARACTERISTICS**

| **No.** | **Questions** | **Answers** |
| --- | --- | --- |
|  | Before your pregnancy, what is your main occupation? | 1. Agriculture/forestry/animal husbandry/fisheries (e.g. farmers, hunters, herders, fishermen, etc.) 2. Production/transportation/equipment operator (e.g. driver, operator, welder, etc.) 3. Service worker (e.g. cook, waitress, hairdressers, saleswoman, laundresses, etc.) 4. Office worker (e.g. secretary, clerk, etc.) 5. Professional and technical personnel (e.g. doctor, teacher, lawyer, architect, editor, athlete, etc.) 6. Administrative personnel (e.g. government official, factory director, manager, etc.) 7. Unemployed 8. Other, please specify ………………….…… |
|  | What is your highest level of education completed? | 1. Primary school 2. Secondary school 3. High school 4. College or University 5. Post-graduate |
|  | What is your family's average monthly income per capita (in Chinese yuan)? | 1. <1500 2. 1500-<3000 3. 3000-<5000 4. 5000-<8000 5. 8000-<10000 6. ≥10000 |
|  | What is your marital status? | 1. Married 2. Single → skip to Q8 3. Divorced/separated→ skip to Q8 4. Widowed→ skip to Q8 |
|  | What is your husband/partner’s main occupation? | 1. Agriculture/forestry/animal husbandry/fisheries (e.g. farmers, hunters, herders, fishermen, etc.) 2. Production/transportation/equipment operator (e.g. driver, operator, welder, etc.) 3. Service worker (e.g. cook, waitress, hairdressers, saleswoman, laundresses, etc.) 4. Office worker (e.g. secretary, clerk, etc.) 5. Professional and technical personnel (e.g. doctor, teacher, lawyer, architect, editor, athlete, etc.) 6. Administrative personnel (e.g. government official, factory director, manager, etc.) 7. Unemployed 8. Other, please specify ………………….…… |
|  | What is the highest level of education your husband/partner completed? | 1. Primary school 2. Secondary school 3. High school 4. College or University 5. Post-graduate 6. Do not know |
|  | What is your husband/partner’s age? | ………….. years |
|  | What was your weight before pregnancy? | □□.□ kg |
|  | Weight now | □□.□ kg |
|  | Height | □□□ cm (measurement) |

**PART III: SMOKING AND ALCOHOL DRINKING**

| **No.** | **Questions** | **Answers** |
| --- | --- | --- |
|  | Did you smoke before this pregnancy? | 1. No 2. Yes, I smoked ………. cigarettes a day on average 3. Quit smoking before becoming pregnant |
|  | Did any family members smoke in front of you before this pregnancy? | 1. No 2. Yes |
|  | Do you smoke during this pregnancy? | 1. No 2. Yes, I smoke ………. cigarettes a day on average |
|  | Do any family members smoke in front of you before this pregnancy? | 1. No 2. Yes |
|  | Did you drink any alcohol products before this pregnancy? | 1. No/occasionally (less than one time per week) 2. Yes (At least one time per week, and last at least half of year) 3. Quit drinking before becoming pregnant |
|  | Do you drink any alcohol products during this pregnancy? | 1. No 2. Occasionally (less than one time per week) 3. Yes (At least one time per week) |

**PART IV: MEDICAL HISTORY**

| **No.** | **Questions** | **Answers** |
| --- | --- | --- |
|  | Do you have diabetes before this pregnancy? | 1. No 2. Yes → 2a: Type I   2b: Type II  2c: gestational diabetes   1. Unknown |
|  | Do you have hypertension before this pregnancy? | 1. No 2. Yes   9. Unknown |
|  | Do you have a history of other chronic diseases? | 1. No 2. Yes → Please specify: …………………………….. |
| **PART V: PREVIOUS INFANT FEEDING PRACTICES AND INFANT FEEDING PLANS** | | |
| **No.** | **Questions** | **Answers** |
|  | After childbirth, will he/she be your first child? | 1. No 2. Yes→ skip to Q24 |
|  | If this is not your first child, did you breastfeed your last child? | 1. No→ skip to Q24 2. Yes |
|  | How long did you breastfeed your last child? | ……………….(months) |
|  | How old was your baby when he or she was first introduced to infant formula? | 1. Never 2. <1 month 3. 1~2 months 4. 2~4 months 5. 4~6 months 6. >6 months |
|  | How do you intend to feed your expecting infant? | 1. Breastmilk only 2. Infant formula only 3. Breastmilk + Infant formula 4. Don’t know 5. Other, please specify……………. |
|  | Does the baby’s father have any preference for how you feed your baby? | 1. He prefers infant formula feeding 2. He prefers breastfeeding 3. He doesn’t mind how I feed my baby 4. Never really discussed the matter with him |
|  | Does your mother have any preference for how you feed your baby? | 1. She prefers infant formula feeding 2. She prefers breastfeeding 3. She doesn’t mind how I feed my baby 4. Never really discussed the matter with her |
|  | Who do you plan to start giving your baby complementary food? (e.g. vegetable puree, infant cereal, egg yolk) | 1. < 2 months 2. 2~3 months 3. 4~6 months 4. 7~9 months 5. > 9 months 6. Don’t know |

**PART VI: Iowa Infant Feeding Attitude Scale**

Mora Adl, Russell DW, Dungy CI, Losch M, Dusdieker L. The Iowa Infant Feeding Attitude Scale: Analysis of Reliability and Validity1. Journal of Applied Social Psychology. 1999;29(11):2362-80.

**THANK YOU FOR YOUR PARTICIPATION**

Participant ID:

**QUESTIONNAIRE 2 (Before discharge)**

**PART I: BASIC INFORMATION**

Date of interview: / / (DD/MM/YYYY)

Interviewer code: □□□□

Participant’s name: ___________________________________________

Phone number (if any change): ______________________________________

Husband/next of kin’s phone number (if any change): __________________________

**PART II: PREGNANCY OUTCOMES (HOSPITAL RECORD)**

| **No.** | **Questions** | **Answers** |
| --- | --- | --- |
|  | Baby’s date of birth | / / (DD/MM/YYYY) |
|  | Gestational age at delivery | ………weeks………days |
|  | Baby’s gender | 1. Male 2. Female |
|  | Birth weight and length | □□□□ grams □□ cm |
|  | Gestational weeks | ………… weeks ………… days |
|  | Delivery method | 1. Caesarean section 2. Vaginal delivery without forceps or suction 3. Vaginal delivery with forceps or suction |
|  | APGAR scores | 1. 1 minute: ……………….. 2. 5 minutes: ………………. |

**PART III: INFANT FEEDING INFORMATION**

1. How have you been feeding your baby since birth?

Breastfeeding only 1

Breastfeeding + (glucose) water 2

Mainly breastfeeding but “topping up” with formula 3

Mainly formula feeding but also breastfeeding 4

Infant formula feeding only→ skip to Q13 5

Other (please specify) _____________________________________________

1. Why did you decide to breastfeed? (**can tick more than one answer**) (Please skip to Q13 if never breastfeed)

|  | NO…1 | YES…2 |
| --- | --- | --- |
| 1. Breastmilk is better for the baby |  |  |
| 1. Breastfeeding is cheaper |  |  |
| 1. Breastfed babies are more intelligent |  |  |
| 1. Breastfeeding helps me lose weight |  |  |
| 1. Breastfeeding is more convenient |  |  |
| 1. Breastfeeding is fashionable |  |  |
| 1. My mother/mother-in-law advised me to breastfeed |  |  |
| 1. The baby’s father wanted me to breastfeed |  |  |
| 1. Other people advised me to breastfeed |  |  |
| 1. Don’t know |  |  |
| 1. Other (please specify) | | |

1. Why did you decide to formula feed your baby? (**can tick more than one answer**) (Please skip to Q14 if never give infant formula)

|  | NO…1 | YES…2 |
| --- | --- | --- |
| 1. No or not enough breastmilk |  |  |
| 1. Breastfeeding is painful |  |  |
| 1. The baby refuses breastfeeding |  |  |
| 1. Inverted nipples |  |  |
| 1. Formula is just as good as breastmilk |  |  |
| 1. Formula is better for the baby |  |  |
| 1. I don’t like breastfeeding |  |  |
| 1. Breastfeeding will make my breasts sag |  |  |
| 1. The baby’s father prefers bottle feeding |  |  |
| 1. My mother or mother-in-law suggested bottle feeding |  |  |
| 1. The baby’s father can help with bottle feeding |  |  |
| 1. Friend or relative suggested bottle feeding |  |  |
| 1. Health worker (e.g. doctor, nurse) suggested bottle feeding |  |  |
| 1. Other (please specify): |  |  |

1. What was your baby’s first feed?

Infant formula 1

Breastmilk (or colostrum) 2

Cow’s milk 3

Glucose water 4

Plain water 5

Other (please specify)

1. How long after birth before you put your baby to the breast?

Within 15 minutes 1

Between 15 and 30 minutes 2

Between 30 minutes and 1 hour 3

Between 1 and 12 hours 4

Between 12 and 24 hours 5

Between 24 and 48 hours 6

Between 48 and 72 hours 7

Baby was given a bottle instead 8

1. How long it took before your milk came in?

Within the same day of the birth 1

The second day after the birth 2

The third day after the birth 3

The fourth day after the birth 4

Still waiting for milk to come in 5

1. Who helped you decide whether you would bottle-feed or breastfeed? (**can tick more than one answer**)

No one, I decided myself 1

The baby’s father 2

My mother or mother-in-law 3

My relatives 4

Friends 5

Doctor 6

Nurse/midwife 7

Other (please specify) ______________________________________

1. Did you have any health problems during this pregnancy? (**can tick more than one answer**)

No 1

Gestational hypertension 2

Gestational diabetes 3

Anaemia 4

Other (please specify) ______________________________________

1. Has the baby been admitted to the neonatal intensive care unit?

No 1

Yes, _______days 2

1. Does the baby have any health problems?

No 1

Yes, please specify__________________ 2

1. Did you remember the messages sent to you during pregnancy?

1. No

2. Yes (list five pieces of information you recall):

a...................................................................

b..................................................................

c.................................................................

d................................................................

e...............................................................

**THANK YOU FOR YOUR PARTICIPATION**

Participant ID:

**QUESTIONNAIRE 3 (1 month postpartum interview)**

**PART I: BASIC INFORMATION**

Date of interview: / / (DD/MM/YYYY)

Interviewer code: □□□□

Participant’s name: ___________________________________________

Phone number (if any change): ______________________________________

Husband/next of kin’s phone number (if any change): __________________________

**PART II: INFANT FEEDING INFORMATION**

1. How did you feed your baby in the last 24 hours?

Breastfeeding only 1

Breastfeeding + (glucose) water 2

Mainly breastfeeding but “topping up” with formula 3

Mainly formula feeding but also breastfeeding 4

Infant formula feeding only 5

Other (please specify) _____________________________________________

1. Have you ever changed the feeding method since discharge?

Yes 1

No→ skip to Q5 2

1. What are the changes? (**can tick more than one answer**)

|  | NO…1 | YES…2 | If yes, how old was the baby when you changed feeding method?  (months) (weeks) | |
| --- | --- | --- | --- | --- |
| 1. Started solids |  |  |  |  |
| 1. Started formula |  |  |  |  |
| 1. Started other milk |  |  |  |  |
| 1. Others: | | |  |  |

1. Who helped you decide to change in feeding method?

I decided myself 1

Baby’s father 2

My mother/ mother-in-law 3

Other family members or relatives 4

Friends 5

Hospital staff 6

Private health workers 7

Others (please specify) _____________________________________________

1. Have you experienced any of the following problems since you started breastfeeding? (**Do not prompt, but can tick more than one answer)**

|  | NO…1 | YES…2 |
| --- | --- | --- |
| 1. I did not experienced any problems related to breastfeeding |  |  |
| 1. Inverted nipples |  |  |
| 1. Cracked or sore nipples |  |  |
| 1. Takes a long time before milk starts flowing at start of feed |  |  |
| 1. Not enough breastmilk |  |  |
| 1. Difficulty expressing milk |  |  |
| 1. Mastitis |  |  |
| 1. Baby not gaining enough weight |  |  |
| 1. Baby has problems sucking |  |  |
| 1. Breast engorged (too full) |  |  |
| 1. Baby refuses breastfeeding |  |  |
| 1. Baby is too tired to breastfeed (e.g. fall asleep at breast) |  |  |
| 1. Trouble positioning and/or attaching the baby to the breast |  |  |
| 1. Feeling that I’m not doing very well at breastfeeding |  |  |
| 1. Baby gets too much milk |  |  |
| 1. Abdominal pain, uterus contraction while breastfeeding |  |  |
| 1. Other (please specify) | | |

1. Does the baby have any health problems?

No 1

Yes, please specify__________________ 2

**THANK YOU FOR YOUR PARTICIPATION**

Participant ID:

**QUESTIONNAIRE 4 (4-month postpartum interview)**

**PART I: BASIC INFORMATION**

Date of interview: / / (DD/MM/YYYY)

Interviewer code: □□□□

Participant’s name: ___________________________________________

Phone number (if any change): ______________________________________

Husband/next of kin’s phone number (if any change): __________________________

**PART II: INFANT FEEDING INFORMATION**

1. How did you feed your baby in the last 24 hours?

Breastfeeding only 1

Breastfeeding + (glucose) water 2

Mainly breastfeeding but “topping up” with formula 3

Mainly formula feeding but also breastfeeding 4

Infant formula feeding only 5

Other (please specify) _____________________________________________

1. Have you ever changed the feeding method since discharge?

Yes 1

No→ skip to Q5 2

1. What are the changes? (**can tick more than one answer**)

|  | NO…1 | YES…2 | If yes, how old was the baby when you changed feeding method?  (months) (weeks) | |
| --- | --- | --- | --- | --- |
| 1. Started solids |  |  |  |  |
| 1. Started formula |  |  |  |  |
| 1. Started other milk |  |  |  |  |
| 1. Others: | | |  |  |

1. Who helped you decide to change in feeding method?

I decided myself 1

Baby’s father 2

My mother/ mother-in-law 3

Other family members or relatives 4

Friends 5

Hospital staff 6

Private health workers 7

Others (please specify) _____________________________________________

1. Have you experienced any of the following problems since you started breastfeeding? **(Do not prompt, but can tick more than one answer)**

|  | NO…1 | YES…2 |
| --- | --- | --- |
| 1. I did not experienced any problems related to breastfeeding |  |  |
| 1. Inverted nipples |  |  |
| 1. Cracked or sore nipples |  |  |
| 1. Mastitis |  |  |
| 1. Takes a long time before milk starts flowing at start of feed |  |  |
| 1. Not enough breastmilk |  |  |
| 1. Difficulty expressing milk |  |  |
| 1. Baby not gaining enough weight |  |  |
| 1. Baby has problems sucking |  |  |
| 1. Breast engorged (too full) |  |  |
| 1. Baby refuses breastfeeding |  |  |
| 1. Baby is too tired to breastfeed (e.g. fall asleep at breast) |  |  |
| 1. Trouble positioning and/or attaching the baby to the breast |  |  |
| 1. Feeling that I’m not doing very well at breastfeeding |  |  |
| 1. Baby gets too much milk |  |  |
| 1. Other (please specify) | | |

1. Have you given complementary foods (solid foods or liquids other than breastmilk or infant formula) to your baby?

No 1

Yes 2

| **Complementary foods** | **When introduced?** |
| --- | --- |
| Vegetable juice | …… months ……… weeks |
| Fruit juice | …… months ……… weeks |
| Rice porridge | …… months ……… weeks |
| Infant cereal | …… months ……… weeks |
| Fruit puree | …… months ……… weeks |
| Vegetable puree | …… months ……… weeks |
| Yolk | …… months ……… weeks |
|  | …… months ……… weeks |
|  | …… months ……… weeks |
|  | …… months ……… weeks |
|  | …… months ……… weeks |
|  | …… months ……… weeks |

**PART III: HEALTH OF MOTHER AND INFANT**

1. Did your baby have any major health problems since the last interview?

No 1

Yes 2

1. What are the health problems? (**can tick more than one answer**)

| **Health problem** | **Number of episodes** |
| --- | --- |
| Vomit, acid reflux |  |
| Diarrhoea |  |
| Upper respiratory tract infection |  |
| Lower respiratory tract infection |  |
| Skin rashes/ roseola infantum |  |
| Stomach ache |  |
| Fever/ Fever of unknown origin |  |
| Jaundice |  |
| Umbilical cord infection |  |
| Blocked tear duct infection |  |
| Constipation |  |
| Injury (e.g. falls, burns): …………………………… |  |
| Others (Please specify): …………………………………. |  |

1. Had your baby been hospitalised since the last interview?

No 1

Yes 2

| **Illness** | **When hospitalised?** | **Length of hospital stay** |
| --- | --- | --- |
|  | …… months ……… weeks | …… weeks ……… days |
|  | …… months ……… weeks | …… weeks ……… days |
|  | …… months ……… weeks | …… weeks ……… days |
|  | …… months ……… weeks | …… weeks ……… days |

1. Did your baby receive any of the following medicines in the past 2 weeks? (Please do not include vitamins or minerals)

| Antibiotics | Yes | No |
| --- | --- | --- |
| Other prescription medicines | Yes | No |
| Non-prescription medicines | Yes | No |

1. Did you experience any health problems since childbirth?

No 1

Yes 2

| **Health problem** | **Number of episodes** |
| --- | --- |
| Urinary Tract Infection |  |
| Diarrhoea |  |
| Upper respiratory tract infection |  |
| Lower respiratory tract infection |  |
| Depression |  |
| Constipation |  |
| Bad headache/ migraine (more than 4 hours) |  |
| Severe back pain |  |
| Accident/injury: ……………………………………. |  |
| Others (Please specify): ………………………………. |  |

1. Had you been hospitalised since childbirth?

No 1

Yes 2

| **Illness** | **When hospitalised?** | **Length of hospital stay** |
| --- | --- | --- |
|  | …… months postpartum | …… weeks ……… days |
|  | ……months postpartum | …… weeks ……… days |
|  | …… months postpartum | …… weeks ……… days |
|  | …… months postpartum | …… weeks ……… days |

1. Have you used any antibiotics in the past three months?

No 1

Yes 2

Don’t know 3

1. Have you returned to work?

No 1

Yes (when) _______________DD/MM/YYYY 2

1. Are you currently exposed to smoke from others?

No 1

Yes 2

1. Did you carefully read the messages sent to you since childbirth?

No 1

Yes, 1-3 messages 2

Yes, 4-6 messages 3

Yes, 7-10 messages 4

Yes, more than 10 messages 5

**PART IV: TERMINATION OF BREASTFEEDING**

**If still breastfeeding no need to complete this section.**

1. How old was your baby when you stopped breastfeeding?

_______months _______weeks

1. Why did you decide to stop breastfeeding?

_____________________________________________________________________

_____________________________________________________________________

1. Did anyone advise you to stop breastfeeding?

No 1

Yes 2 (Who? _______________________________________________)

**THANK YOU FOR YOUR PARTICIPATION**

Participant ID:

**QUESTIONNAIRE 5 (6-month postpartum interview)**

**PART I: BASIC INFORMATION**

Date of interview: / / (DD/MM/YYYY)

Interviewer code: □□□□

Participant’s name: ___________________________________________

Phone number (if any change): ______________________________________

Husband/next of kin’s phone number (if any change): __________________________

**PART II: INFANT FEEDING INFORMATION**

1. How did you feed your baby in the last 24 hours?

Breastfeeding only 1

Breastfeeding + (glucose) water 2

Mainly breastfeeding but “topping up” with formula 3

Mainly formula feeding but also breastfeeding 4

Infant formula feeding only 5

Other (please specify) _____________________________________________

1. Have you ever changed the feeding method since discharge?

Yes 1

No→ skip to Q5 2

1. What are the changes? (**can tick more than one answer**)

|  | NO…1 | YES…2 | If yes, how old was the baby when you changed feeding method?  (months) (weeks) | |
| --- | --- | --- | --- | --- |
| 1. Started solids |  |  |  |  |
| 1. Started formula |  |  |  |  |
| 1. Started other milk |  |  |  |  |
| 1. Others: | | |  |  |

1. Who helped you decide to change in feeding method?

I decided myself 1

Baby’s father 2

My mother/ mother-in-law 3

Other family members or relatives 4

Friends 5

Hospital staff 6

Private health workers 7

Others (please specify) _____________________________________________

1. Have you experienced any of the following problems since you started breastfeeding? **(Do not prompt, but can tick more than one answer)**

|  | NO…1 | YES…2 |
| --- | --- | --- |
| 1. I did not experienced any problems related to breastfeeding |  |  |
| 1. Inverted nipples |  |  |
| 1. Cracked or sore nipples |  |  |
| 1. Mastitis |  |  |
| 1. Takes a long time before milk starts flowing at start of feed |  |  |
| 1. Not enough breastmilk |  |  |
| 1. Difficulty expressing milk |  |  |
| 1. Baby not gaining enough weight |  |  |
| 1. Baby has problems sucking |  |  |
| 1. Breast engorged (too full) |  |  |
| 1. Baby refuses breastfeeding |  |  |
| 1. Baby is too tired to breastfeed (e.g. fall asleep at breast) |  |  |
| 1. Trouble positioning and/or attaching the baby to the breast |  |  |
| 1. Feeling that I’m not doing very well at breastfeeding |  |  |
| 1. Baby gets too much milk |  |  |
| 1. Other (please specify) | | |

1. Have you given complementary foods (solid foods or liquids other than breastmilk or infant formula) to your baby?

No 1

Yes 2

| **Complementary foods** | **When introduced?** |
| --- | --- |
| Vegetable juice | …… months ……… weeks |
| Fruit juice | …… months ……… weeks |
| Rice porridge | …… months ……… weeks |
| Infant cereal | …… months ……… weeks |
| Fruit puree | …… months ……… weeks |
| Vegetable puree | …… months ……… weeks |
| Yolk | …… months ……… weeks |
|  | …… months ……… weeks |
|  | …… months ……… weeks |
|  | …… months ……… weeks |
|  | …… months ……… weeks |
|  | …… months ……… weeks |

**PART III: HEALTH OF MOTHER AND INFANT**

1. Did your baby have any major health problems since the last interview?

No 1

Yes 2

1. What are the health problems? **(can tick more than one answer)**

| **Health problem** | **Number of episodes** |
| --- | --- |
| Vomit, acid reflux |  |
| Diarrhoea |  |
| Upper respiratory tract infection |  |
| Lower respiratory tract infection |  |
| Skin rashes/ roseola infantum |  |
| Stomach ache |  |
| Fever/ Fever of unknown origin |  |
| Umbilical cord infection |  |
| Blocked tear duct infection |  |
| Constipation |  |
| Injury (e.g. falls, burns): …………………………… |  |
| Others (Please specify): …………………………………. |  |

1. Had your baby been hospitalised since the last interview?

No 1

Yes 2

| **Illness** | **When hospitalised?** | **Length of hospital stay** |
| --- | --- | --- |
|  | …… months ……… weeks | …… weeks ……… days |
|  | …… months ……… weeks | …… weeks ……… days |
|  | …… months ……… weeks | …… weeks ……… days |
|  | …… months ……… weeks | …… weeks ……… days |

1. Did your baby receive any of the following medicines in the past 2 weeks? (Please do not include vitamins or minerals)

| Antibiotics | Yes | No |
| --- | --- | --- |
| Other prescription medicines | Yes | No |
| Non-prescription medicines | Yes | No |

1. Did you experience any health problems in the past 2 months?

No 1

Yes 2

| **Health problem** | **Number of episodes** |
| --- | --- |
| Urinary Tract Infection |  |
| Diarrhoea |  |
| Upper respiratory tract infection |  |
| Lower respiratory tract infection |  |
| Depression |  |
| Constipation |  |
| Bad headache/ migraine (more than 4 hours) |  |
| Severe back pain |  |
| Accident/injury: ……………………………………. |  |
| Others (Please specify): ………………………………. |  |

1. Had you been hospitalised in the past 2 months?

No 1

Yes 2

| **Illness** | **When hospitalised?** | **Length of hospital stay** |
| --- | --- | --- |
|  | …… months postpartum | …… weeks ……… days |
|  | ……months postpartum | …… weeks ……… days |
|  | …… months postpartum | …… weeks ……… days |
|  | …… months postpartum | …… weeks ……… days |

1. Have you used any antibiotics in the past 2 months?

No 1

Yes 2

Don’t know 3

1. Have you returned to work?

No 1

Yes (when) _______________DD/MM/YYYY 2

1. Are you currently exposed to smoke from others?

No 1

Yes 2

1. Did you carefully read the messages sent to you in the past 2 months?

No 1

Yes, 1-3 messages 2

Yes, 4-6 messages 3

Yes, 7-10 messages 4

**PART IV: TERMINATION OF BREASTFEEDING**

**If still breastfeeding no need to complete this section.**

1. How old was your baby when you stopped breastfeeding?

_______months _______weeks

1. Why did you decide to stop breastfeeding?

_____________________________________________________________________

_____________________________________________________________________

1. Did anyone advise you to stop breastfeeding?

No 1

Yes 2 (Who? _______________________________________________)

**THANK YOU FOR YOUR PARTICIPATION**
