# Supplementary material for: WeChat-based intervention to support breastfeeding for Chinese mothers: protocol of a randomised controlled trial
Source: BMC Med Inform Decis Mak. 2020 Nov 19;20:300. doi: 10.1186/s12911-020-01322-8 (PMC7676472; doi:10.1186/s12911-020-01322-8)
Supplement: Supplementary file 2 — Additional file 2: Intervention messages. [file 12911_2020_1322_MOESM2_ESM.docx]

1. **Before delivery**
2. **Intervention messages**

|  | **Theme** | **Message** |
| --- | --- | --- |
| 1 | WHO’s recommendation on exclusive breastfeeding | The benefits of breastfeeding for both mother and infant have been consistently demonstrated. Worldwide, many health organizations recommend and support breastfeeding. For instance, the WHO recommends exclusive breastfeeding of infants for the first 6 months of life (no other food/drinks). |
| 2 | Breastmilk’s composition (Nutrients) | Breastmilk has all the nutrients the baby needs and is the ideal food for newborns and infants. Breastmilk provides all food and water a baby needs until about 6 months. The composition of breastmilk changes to meet the specific needs of the infant. |
| 3 | Breastmilk’s composition (Antibodies) | Breastmilk contains antibodies that help infants to fight infections. Breastmilk will prevent your child to have diarrhoea or pneumonia, and helps for a quicker recovery if ill. |
| 4 | Long-term benefits | Research data suggest that breastfeeding is associated with higher IQ and better academic performance, when compared with infant formula feeding. |
| 5 | To avoid alcohol | Alcohol in the blood of pregnant mother passes to the baby through the placenta. Alcohol, even small amounts, in the fetus can adversely affect their brain development. It is wise to avoid drinking any alcoholic drink during the whole pregnancy. |
| 6 | Long-term benefits | Adolescents and adults who were breastfed as babies are less likely to be overweight or obese & less type-2 diabetes. |
| 7 | Benefits for mothers | Breastfeeding will help you reduce your weight after delivery & return to your shape. If you breastfeed, your chance of having breast or ovarian cancer later in life will be reduced. |
| 8 | Infant formula will never be superior or identical to breastmilk. | Don't believe formula milk's advertisements. Breast milk is the best (formula milk is cow’s milk – great for baby cows, not baby humans). Although the goal of infant formula is to duplicate the composition of breastmilk, no product will be identical to breastmilk. |
| 9 | Colostrum | Colostrum, yellowish milk, is high in protein and antibodies. Colostrum is of great importance in preventing your child from being sick. In the first few days, it is more important to let your baby suckle frequently than to nurse for long periods of time. During these days, there is not much breastmilk, and the breasts are soft, which is suitable for your baby to learn to latch. |
| 10 | Anaesthetic and analgesic drugs (C-section surgery) | Delivery method has little impact on breastmilk secretion. Give your baby colostrum, soon after deliver whether you deliver normal or by operation. Frequent suckling helps you produce adequate milk. For a C-section mother, the anaesthetic and analgesic drugs present in her blood after the operation are safe for her suckling infant. |

1. **Control messages**

|  | **Theme** | **Message** |
| --- | --- | --- |
| 1 | Balanced diet | A healthy and balanced diet is of great importance to both mother and baby. Eat a large variety of food, including grains, vegetables, fruit, protein foods (meat, fish, eggs), and dairy to get the nutrients you need every day. Limit the intake of food and drinks high in sugar, fat, and calories. |
| 2 | Smoking and second-hand smoking | Smoking during pregnancy exposes the unborn infant to an increased risk of health problems. In fact, second-hand smoking can also have detrimental effects on the health of your baby. The harmful chemicals present in second-hand smoke can be passed to the fetus. |
| 3 | Preparing for labour | It’s natural to start thinking about giving birth and plan ahead as much as you can. Attend antenatal classes. |
| 4 | Breastmilk’s composition (Nutrients) | Breastmilk has all the nutrients the baby needs and is the ideal food for newborns and infants. Breastmilk provides all food and water a baby needs until about 6 months. The composition of breastmilk changes to meet the specific needs of the infant. |
| 5 | To avoid alcohol | Alcohol in the blood of pregnant mother passes to the baby through the placenta. Alcohol, even small amounts, in the fetus can adversely affect their brain development. |
| 6 | Exercise | Regular exercise during pregnancy may help you sleep better, improve your mood, reduce backaches and constipation, and may improve your ability to cope with labour. A healthy pregnant woman is recommended to have approximately 30 minutes of moderate intensity physical activity, such as brisk walking and indoor stationary cycling. |
| 7 | Gestational hypertension | If you experience some of the symptoms below, go to health clinic. You can have high blood pressure. The symptoms include: persistent severe headache, swelling legs and face, blurred vision, nausea or vomiting, and abdominal pain. |
| 8 | Heartburn | Heartburn is a common complaint during pregnancy. The growing uterus which puts pressure on the stomach. Pregnancy hormones relaxing the valve between stomach and oesophagus can allow stomach acid to reflux into oesophagus, causing heartburn. Eat several small meals each day instead of three large ones, eat slowly, don’t lie down immediately after eating, avoid spicy, greasy and fatty foods. |
| 9 | Signs of labour | It is time to go to the hospital when you experience either of the following: waters broke with a trickle or a gush; contractions become stronger, get regular, last longer, are closer together as time progresses, and do not stop when you change your position or relax. |
| 10 | Infant formula will never be superior or identical to breastmilk. | Don't believe formula milk's advertisements. Breast milk is the best (formula milk is cow’s milk – great for baby cows, not baby humans). Although the goal of infant formula is to duplicate the composition of breastmilk, no product will be identical to breastmilk. |

1. **After delivery**
2. **Intervention messages**

|  | **Theme** | **Message** |
| --- | --- | --- |
| 0 | Congratulations on your new baby | Congratulations on your new baby! We will send you messages on infant feeding and parental care in the next half a year. |
| 1 | Correct positioning | Correct positioning can help the baby latch onto the breast effectively. When holding your baby, make sure the baby’s head and body are in a straight line, and make sure the baby is facing the breast with the baby’s nose opposite your nipple and chin touching the breast. |
| 2 | Criteria to know breastmilk is adequate or not | When you are breastfeeding, listen for gulping sounds so that you know your baby is actually swallowing milk. The baby's jaw should move in a slow, steady manner as he/she sucks and swallows. If your baby is getting enough breastmilk, he/she has at least 6 wet diapers a day (after day 3) and the urine is the diapers should be pale yellow-coloured. |
| 3 | Do not give water to a breastfeeding baby for the first 6 months of life | Breastfed babies do not need water before they are six months old, even when it is hot. Breastmilk is more than 80% water. When breastfeeding, the breastmilk provides the baby all the water he/she needs. |
| 4 | Immunization | Immunizations is an important way to protect your baby’s health. Many serious diseases, some of which can be life-threatening, are now vaccine-preventable. This had led to a much reduced morbidity rate in children. |
| 5 | Breastfeeding with a flu | If you have a flu while breastfeeding, all possible precautions should be taken to avoid spreading the virus to your baby while continuing to provide breastmilk to him/her. The illness will not be spread to your baby through breastmilk. In fact, it will contain antibodies that can help protect your baby. Consult your doctor before taking any over-the-counter flu medicines. |
| 6 | Breastmilk production | Almost all mothers can produce adequate milk to feed their infants, unless position is not correct or feeding frequency is not enough. The more your infant sucks correctly, the more milk you produce and deliver, until you have both negotiated the proper balance. |
| 7 | Infant’s growth curve | Measuring and recording your baby’s weight and length on a regular basis will help you understand your baby’s development. In general, babies should be somewhere between the 97^th^ and 3^rd^ percentile. Besides the age and sex specific points, attentions should also be given to the trend of growth curve as growth is a continuous process. |
| 8 | Complementary feeding | Around the age of 6 months, an infant’s need for energy and nutrients starts to exceed what is provided by breastmilk, and complementary foods are necessary to meet those needs. Therefore, infants are recommended to start receiving complementary foods at 6 months of age. An infant of this age is also developmentally ready to eat purred, mashed and semi-solid foods. |
| 9 | Breastfeeding and birth control | Breastfeeding can be used as a method of birth control. But three conditions must be met to ensure its effectiveness (still with a failure rate of 2%): your baby must be 6 months of age or younger; your baby receives breastmilk only; and you must not have a period. It is important to remember that breastfeeding must be maintained with both day and night feeding. If the intervals between feedings go longer, you must use an additional form of birth control if you do not want to get pregnant. |
| 10 | Safe sleep for infant | In the first year, babies should sleep on their backs for all sleep times. But if your baby is comfortable rolling back to tummy and tummy to back, then you do not have to return him/her to the back. Bed-sharing is not recommended for any babies. |
| 11 | Breastfeeding preparedness before returning to work | Start practice pumping or expressing milk one week before returning to work. This also allows you to store milk that the care provider can feed to your baby. In addition to breast pumps and/or milk collection kits, make sure to have an ice pack for safe storage of breastmilk on the way home from work. |
| 12 | Breastfeeding and exercise | Regular exercise is safe and healthy for breastfeeding mothers. Although the lactic acid in breastmilk can increase after high intensity exercise, mild and moderate exercise does not cause lactic acid to increase and does not affect a baby taking the milk. There is no evidence that breastmilk with increased lactic acid harms a baby. |
| 13 | Storage of expressed breastmilk | Freshly expressed breastmilk can be stored at room temperature (25°C or colder) for no more than 4 hours. It can be kept in a cooler with an ice pack for up to 24 hours (15°C); can be refrigerated (4°C) for up to 4 days. If it is not going to be used in 48~72 hours, it is best to freeze it. In the freezer (-18°C or colder), breastmilk is the best within 6 months. |
| 14 | Safe thawing of breastmilk | Do not thaw or heat breastmilk in a microwave. Microwaving creates hot spots which can burn a baby’s mouth. You can thaw your breastmilk by holding the frozen bottle or bag under lukewarm running water. You can also thaw it in the refrigerator or in a container of warm water. Always thaw the oldest breastmilk first. |
| 15 | Complementary feeding | It is recommended to start complementary feeding at around 6 months old. Introduction of complementary foods too early may significantly reduce your milk supply, and possibly impose an adverse effect on the nutritional status of your infant. |
| 16 | How to increase breastmilk supply | The more milk is removed from the breast, and the more milk is made. Your baby at the breast sucking is the most effective way to remove milk from your breast, but otherwise use a hand or electric pump. If you are away from your baby, remember to pump at the same times your baby would normally be nursing. |
| Last | Closing remarks | Thank you once again for participating in our study. We wish you and your baby much health and happiness! |

1. **Control messages**

|  | **Theme** | **Message** |
| --- | --- | --- |
| 0 | Congratulations on your new baby | Congratulations on your new baby! We will send you messages on infant feeding and parental care in the next half a year. |
| 1 | Immunization | Immunizations is an important way to protect your baby’s health. Many serious diseases, some of which can be life-threatening, are now vaccine-preventable. This had led to a much reduced morbidity rate in children. |
| 2 | Do not give water to a breastfeeding baby for the first 6 months of life | Breastfed babies do not need water before they are six months old, even when it is hot. Breastmilk is more than 80% water. When breastfeeding, the breastmilk provides the baby all the water he/she needs. |
| 3 | Healthy lifestyle after birth | Regardless of breastfeeding or not, you need to eat a healthy diet to help you recover. Focus on a diet with plenty of fresh vegetables and fruits, balanced with proteins and carbohydrates. Cut out high-fat snacks, such as crisps, biscuits and cakes. |
| 4 | Clothing tips | In hot weather (>24°C), a good rule is to dress the baby in one more layer of clothing that you are wearing to be comfortable in the same environment. In colder weather (<24°C), it's generally best to dress your baby in an undershirt and diapers, covered by pajamas or a dressing gown, and then wrap him or her in a receiving blanket. |
| 5 | Responding to baby’s cries | You will not spoil a baby by holding her/him while she/he is crying. If leaving a baby to cry, it will be stressful and may affect baby’s health and development. Pay close attention to your baby’s different cries. You may soon be able to identify his/her needs by the way he/she cries. |
| 6 | Infant safety | Babies are too young to take care of themselves. Parents play an important role in keeping their children safe. Do not leave your baby alone on changing tables, beds, sofas, or chairs. Never carry your baby and hot liquids or foods at the same time. |
| 7 | Postpartum mental health | It is easy to forget that happy and positive events can cause stress. For instance, marriage, pregnancy and childbirth are happy events, but they can also turn into stress, anxiety, and even depression. Due to hormonal changes that happen after a baby is born and changes such as the loss of sleep and increased stress that come with taking care of a newborn baby, many new mothers feel anxious, sad, frustrated, tried, and overwhelmed in the first few weeks after delivery. It is normal to feel emotional when you are stressed. Sometimes the symptoms may be involuntary or ignored by family members and friends. As a result, appropriate help may not be given timely and this may cause personal and family problems. These feelings may get better within a few weeks. But for some women, the feelings are very strong or do not get better. If they do not go away after about two weeks or make it hard for you to take care of your baby, get help right away. You can start by talking to your health care provider. |
| 8 | Infant’s growth curve | Measuring and recording your baby’s weight and length on a regular basis will help you understand your baby’s development. In general, babies should be somewhere between the 97^th^ and 3^rd^ percentile. Besides the age and sex specific points, attentions should also be given to the trend of growth curve as growth is a continuous process. |
| 9 | Breastmilk production | Almost all mothers can produce adequate milk to feed their infants, unless position is not correct or feeding frequency is not enough. The more your infant sucks correctly, the more milk you produce and deliver, until you have both negotiated the proper balance. |
| 10 | Infant safety | Never leave your baby alone in the bath, even for a moment. If you have to answer the phone or doorbell while your baby is in the tub, wrap your baby in a tower and take him/her with you. |
| 11 | Infant sleep | Newborn babies sleep much of the time, but often in stretches of a few hours at a time. As a baby grows, the total amount of sleep slowly decreases, and the length of night-time sleep increases. Many babies sleep at least five hours at a time by age 3 to 4 months. But every baby is different. Your baby will have her own pattern of waking and sleeping, and it’s unlikely to be the same as other babies you know. |
| 12 | Keep a smoke-free environment for your baby | Second-hand smoke is a common indoor pollutant at homes. It contains thousands of chemicals, many of which are dangerous. Children exposed to second-hand smoke can have more health problems, including ear infections, coughs, colds and respiratory problems, such as bronchitis and pneumonia. |
| 13 | Hair loss | Hair loss occurring in the first six months of life is normal and to be expected. The baby hair falls out and is replaced by permanent hair. At 3 to 6 months old, it is very common that a baby loses his/her hair where he/she rubs his/her scalp against the mattress or as a result of a head banging habit. The hair grows back once the baby starts sitting up. |
| 14 | Car seat safety | Using a car seat is the best way to protect your baby when in a car. Babies are at greater risk of injury in crashes. Always put your infant in a rear-facing child safety seat in the back of your car. |
| 15 | Safe sleep for infant | In the first year, babies should sleep on their backs for all sleep times. But if your baby is comfortable rolling back to tummy and tummy to back, then you do not have to return him/her to the back. Bed-sharing is not recommended for any babies. |
| 16 | Complementary feeding | It is recommended to start complementary feeding at around 6 months old. Introduction of complementary foods too early may significantly reduce your milk supply, and possibly impose an adverse effect on the nutritional status of your infant. |
| Last | Closing remarks | Thank you once again for participating in our study. We wish you and your baby much health and happiness! |
